# Effect of film thickness and evaporation rate on co-evaporated SnSe thin films for photovoltaic applications

**DOI:** 10.1039/d0ra01749c

**Published:** 2020-04-29

**Authors:** Zeng Li, Yixin Guo, Fei Zhao, Chengqi Nie, Hongkai Li, Jingyu Shi, Xiaohui Liu, Jinchun Jiang, Shaohua Zuo

**Affiliations:** Engineering Research Center of Nanoelectronic Integration and Advanced Equipment, Ministry of Education, School of Communication and Electronic Engineering, East China Normal University China; Engineering Research Center of Nanoelectronic Integration and Advanced Equipment, Ministry of Education, School of Physics and Electronic Science, East China Normal University China; School of Computer and Information, Hohai University China

## Abstract

SnSe thin films were deposited by a co-evaporation method with different film thicknesses and evaporation rates. A device with a structure of soda-lime glass/Mo/SnSe/CdS/i-ZnO/ITO/Ni/Al was fabricated. Device efficiency was improved from 0.18% to 1.02% by a film thickness of 1.3 μm and evaporation rate of 2.5 Å S^−1^*via* augmentation of short-circuit current density and open-circuit voltage. Properties (electrical, optical, structural) and scanning electron microscopy measurements were compared for samples. A SnSe thin-film solar cell prepared with a film thickness of 1.3 μm and evaporation rate of 2.5 Å S^−1^ had the highest electron mobility, better crystalline properties, and larger grain size compared with the other solar cells prepared. These data can be used to guide growth of high-quality SnSe thin films, and contribute to development of efficient SnSe thin-film solar cells using an evaporation-based method.

## Abstract 2

SnSe thin films were deposited by a co-evaporation method with different film thicknesses and evaporation rates.

## Graphical abstract

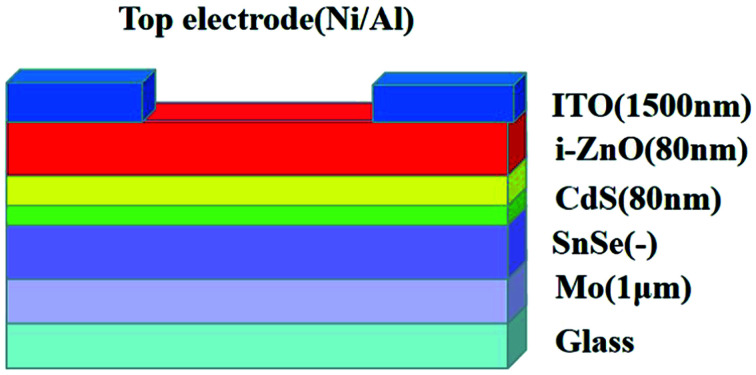

## 1. Introduction

The extensive electronic transitions between delocalized and localized energy-band states ensure that chalcogenides based on transition metals have good optical and electrical properties. These attributes provide considerable convenience for solving the problem of energy shortages in different fields. Due to the excellent electronic and optical properties of binary compounds in transition metals, many scholars have conducted in-depth research in photovoltaic and solar-cell systems.^1–5^

Among binary compounds in transition metals, SnSe has great application prospects because SnSe has an orthorhombic crystal structure,^6^ which is suitable for solar cells. SnSe is a p-type semiconductor and its high absorption coefficient (10^5^ cm^−1^) ensures that solar radiation is absorbed almost completely.^7,8^ Besides, the maximum theoretical efficiency of 32%, rich content, and non-toxic nature in earth of SnSe thin-film solar cells make them suitable for photovoltaic applications.^7,8^ Four solid phases exist in a Sn–Se system (Se, Sn, SnSe and SnSe_2_) to make it easier to control the phase composition during growth of SnSe compounds.^9,10^

SnSe thin films can be obtained by various growth methods: chemical-bath deposition,^7^ thermal evaporation,^11^ brush plates,^12^ two-stage processes,^13^ electro-deposition,^14^ and spray pyrolysis.^15^ The vacuum method is important because of its low cost and low complexity of material synthesis. Despite the extraordinary properties it shows for photovoltaic applications, only a few reports have been published on its layers and device studies, which have poor efficiency (<1%).^16–20^

In the present study, Sn and Se were evaporated simultaneously in a vacuum by thermal evaporation to form SnSe thin films. This method dictates that film formation is dependent upon the substrate temperature, evaporation rate, film thickness, and purity of the material. To improve optical and electrical properties, the evaporation rate and thickness of the SnSe thin film are necessary for the synthesis of SnSe thin films. However, several studies have not paid attention to the influence of the evaporation rate and film thickness on the structure, surface morphology, optical properties and electrical properties of SnSe thin films, which limits the power-conversion efficiency of solar cells made from SnSe thin films.

In this work, to enhance optical and electrical properties, SnSe thin films were prepared under various evaporation rates and film thicknesses by co-evaporation. The effects of evaporation rate and film thickness on the properties of SnSe thin films were studied. Finally, SnSe thin-film solar cells with a soda-lime glass (SLG)/Mo/SnSe/CdS/i-ZnO/ITO/Ni/Al structure were prepared and showed optimal conversion efficiency of 1.02% at an evaporation rate of 2.5 Å S^−1^ and film thickness of 1.3 μm.

## 2. Experimental

### 2.1 Deposition of thin films

**Fig. 1:**
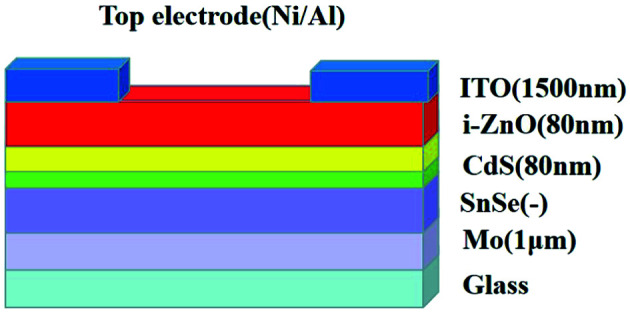
Structure of the SnSe thin-film solar cell.

Sn (purity = 99.999%) and Se (purity = 99.999%) were used to deposit SnSe thin films by co-evaporation. All SnSe thin films were deposited on a bare glass substrate and molybdenum-coated soda-lime glass substrate. The evaporation rate was controlled to 1, 2.5, 5 Å S^−1^ to analyze the effects of evaporation rate on the properties of SnSe thin films. To investigate the influence of film thickness on the properties of SnSe thin films, film thickness was set to 0.6, 0.9, and 1.3 μm. The experiments mentioned above were executed in a vacuum and an operating pressure of 10^−5^ torr. Besides, all films were deposited at 500 °C and on the same substrate type (bare glass). SnSe thin films prepared on a bare glass substrate were used for optical characteristics, electrical characteristics, structural analyses, and analyses of surface morphology. SnSe thin films prepared on the SLG substrate were integrated into the solar cell. The SnSe thin-film solar cells were fabricated by depositing a 70 nm CdS buffer layer in a pure argon atmosphere at 0.45 Pa, room temperature for 5 min, and deposition power of 100 W with the rf-sputtering method on SnSe absorber films prepared on co-evaporation, followed by deposition of a 70 nm i-ZnO layer and 1500 nm ITO layer. Using an intrinsic ZnO target, deposition of the i-ZnO layer was by rf-sputtering at room temperature for 5 min, deposition power of 100 W, and in an atmosphere of pure argon at 0.45 Pa. The ITO layer was deposited by rf-sputtering using an ITO target at room temperature for 15 min, deposition power of 100 W, and in a pure argon atmosphere at 0.5 Pa. Nickel (60 nm) and aluminum (400 nm) top contacts were obtained by deposition using electron-beam evaporation. The total area of each SnSe solar cell was ∼0.42 cm^2^ and etching, element doping, diffusion barrier or anti-reflection coating were not used in the device fabrication. The structure of the SnSe thin-film solar cell in this research is shown as Fig. 1.

### 2.2 Characterization

Raman spectroscopy was undertaken using a Raman spectrometer (Jobin-Yvon T64000). The structure was observed by X-ray diffraction (XRD) using a Rigaku D/max 2550 V system with Cu Kα radiation. The atomic ratio of Sn/Se was estimated by X-ray fluorescence (XRF) using a Shimadzu EDX-7000 setup. The optical parameters were taken by Cary5000 UV-VIS-NIR spectrophotometer. Surface morphology was investigated by scanning electron microscopy (SEM) using a JEOL setup. Current–voltage (*J*–*V*) characteristics were studied using a solar simulator (Xe lamp; Newport) with a source meter (2400; Keithley) at 100 mW cm^−2^ and AM 1.5 G illumination. The electrical properties of the films were determined by measurement of the Hall effect (HMS3000; Ecopia).

## 3. Results and discussion

### 3.1 Crystalline structure

**Fig. 2:**
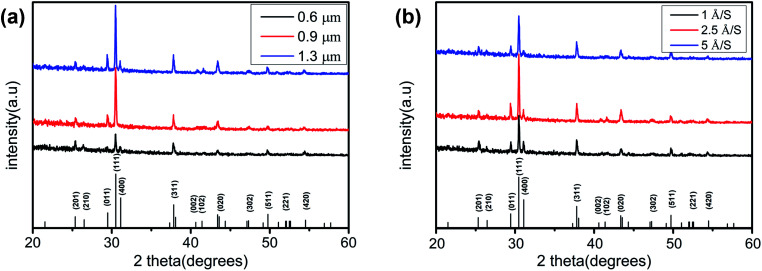
(a) XRD patterns of SnSe thin films with different thicknesses at an evaporation rate of 2.5 Å S^−1^; (b) XRD patterns of SnSe thin films with different evaporation rates at a thickness of 1.3 μm.

Table 1 shows the composition of SnSe thin films measured by XRF. The Sn/Se atomic ratio of all samples was close to 1. Fig. 2a shows the XRD patterns of the SnSe thin films at a film thickness of 0.6, 0.9, and 1.3 μm with an evaporation rate of 2.5 Å S^−1^ to study the crystalline structure of these films. Obvious diffraction peaks at 2*θ* = 25.317°, 26.450°, 29.425°, 30.462°, 31.081°, 37.784°, 40.585°, 41.364°, 43.538°, 47.255°, 49.710°, 51.043° and 54.475° were noted, which are consistent with SnSe (PDF#48-1224). Compared with XRD patterns with a film thickness of 0.6 and 0.9 μm, almost all diffraction peaks were sharper and stronger at a film thickness of 1.3 μm. This observation was because of a reduction in stacking faults and lattice defects as the film thickness increased. The (111) diffraction peak was significantly stronger than the peaks at other diffractions, indicating that the SnSe thin film had a preferential orientation of the (111) phase. The superiority of the (111) peak helps to improve the efficiency of solar cells.^21^ Initially conclusions from Fig. 2a suggest that the SnSe thin film with a thickness of 1.3 μm was superior to SnSe thin films with a film of 0.6 or 0.9 μm. Fig. 2b shows the XRD patterns of the SnSe thin films at an evaporation rate of 1, 2.5, and 5 Å S^−1^ with a film thickness of 1.3 μm. Obvious diffraction peaks at 2*θ* = 25.317°, 26.450°, 29.425°, 30.462°, 31.081°, 37.784°, 40.585°, 41.364°, 43.538°, 47.255°, 49.710°, 51.043° and 54.475° were documented. Compared with the XRD patterns with an evaporation rate of 1 or 5 Å S^−1^, the evaporation rate of 2.5 Å S^−1^ exhibited stronger diffraction peaks, especially the (111) diffraction peak. Besides, the (111) diffraction peak was sharper and stronger than other peaks among all the diffraction peaks and all samples. Combined with the XRD data shown in Fig. 2a and b, SnSe thin films prepared at an evaporation rate of 2.5 Å S^−1^ and film thickness of 1.3 μm may be more suitable for photovoltaic devices.

**Table 1:** Elemental composition of SnSe films

Thickness (μm)	Evaporation rate (Å S^−1^)	Sn (at%)	Se (at%)	Sn/Se ratio
0.6	2.5	51.462	48.538	1.06
0.9	2.5	51.782	48.218	1.07
1.3	2.5	50.224	49.776	1.01
1.3	1	50.682	49.318	1.02
1.3	5	50.416	49.584	1.01

The lattice parameters of the crystal were calculated using the following formula:
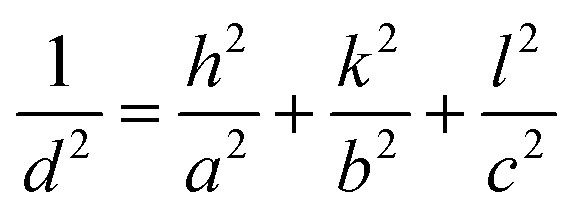
where *d* is the distance between planes, *h*, *k*, *l* are Miller indices, and *a*, *b*, *c* are lattice constants. The lattice constants of these SnSe thin films had the following values: *a* = 11.50 Å, *b* = 4.15 Å, and *c* = 4.44 Å. These values are in good agreement with those in the Joint Committee on Powder Diffraction Standards (JCPDS) database, as well as the literature for SnSe thin films.^22^ Detailed structural parameters of all films are presented in Table 2.

**Table 2:** Structural parameters of SnSe thin films. “*h k l*” denotes Miller indices, and *d* is the distance between planes

SnSe thin films deposited with different thicknesses at the evaporation rate of 2.5 Å S^−1^			SnSe thin films deposited with different evaporation rates at the thickness of 1.3 μm		
2*θ* (°)	(*h k l*)	*d* (Å)	2*θ* (°)	(*h k l*)	*d* (Å)
25.317	(2 0 1)	3.5150	25.317	(2 0 1)	3.5150
26.450	(2 1 0)	3.3670	26.450	(2 1 0)	3.3670
29.425	(0 1 1)	3.0330	29.425	(0 1 1)	3.0330
30.462	(1 1 1)	2.9320	30.462	(1 1 1)	2.9320
31.081	(4 0 0)	2.8750	31.081	(4 0 0)	2.8750
37.784	(3 1 1)	2.3790	37.784	(3 1 1)	2.3790
40.585	(0 0 2)	2.2210	40.585	(0 0 2)	2.2210
41.364	(1 0 2)	2.1810	41.364	(1 0 2)	2.1810
43.538	(0 2 0)	2.0770	43.538	(0 2 0)	2.0770
47.255	(3 0 2)	1.9219	47.255	(3 0 2)	1.9219
49.710	(5 1 1)	1.8326	49.710	(5 1 1)	1.8326
51.043	(2 2 1)	1.7878	51.043	(2 2 1)	1.7878

### 3.2 Morphology

**Fig. 3:**
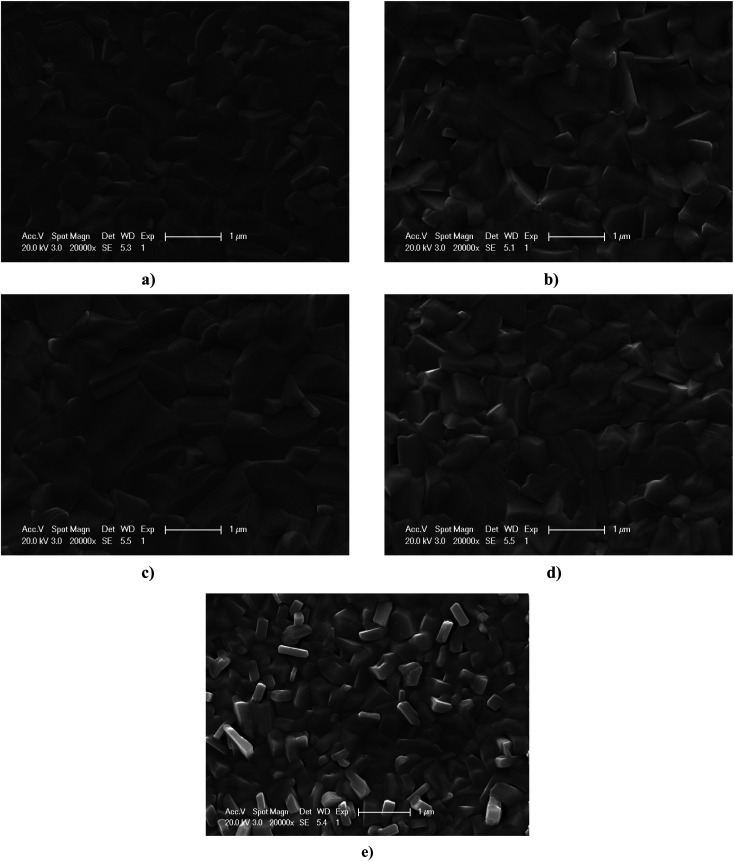
SEM images of SnSe thin films under different deposition conditions: (a) film thickness of 0.6 μm and evaporation rate of 2.5 Å S^−1^; (b) film thickness of 0.9 μm and evaporation rate of 2.5 Å S^−1^; (c) film thickness of 1.3 μm and evaporation rate of 2.5 Å S^−1^; (d) film thickness of 1.3 μm and evaporation rate of 1 Å S^−1^; (e) film thickness of 1.3 μm and evaporation rate of 5 Å S^−1^.

Fig. 3 shows the surface SEM images of SnSe thin films deposited under different conditions. Although the microcrystals for all SnSe films were distributed uniformly over the film surface, the microstructure (crystallinity and grain size) of the samples was dependent upon the deposition conditions. Grain size could be enhanced by changing the thickness of the SnSe thin film. Crystallinity and grain size could also be altered by changing the evaporation rate. The grain size of SnSe thin films with a thickness of 1.3 μm and evaporation rate of 2.5 Å S^−1^ was larger than that in other SnSe thin films, which indicated enhanced grain growth and better crystallinity using this deposition condition. Usually, it is important to acquire absorber films with better crystallinity or large grain size for high energy-conversion efficiency because smaller grains lead to recombination, which results in a decrease of open circuit voltage (*V*_oc_) and reduction in current.^23^

### 3.3 Optical properties

**Fig. 4:**
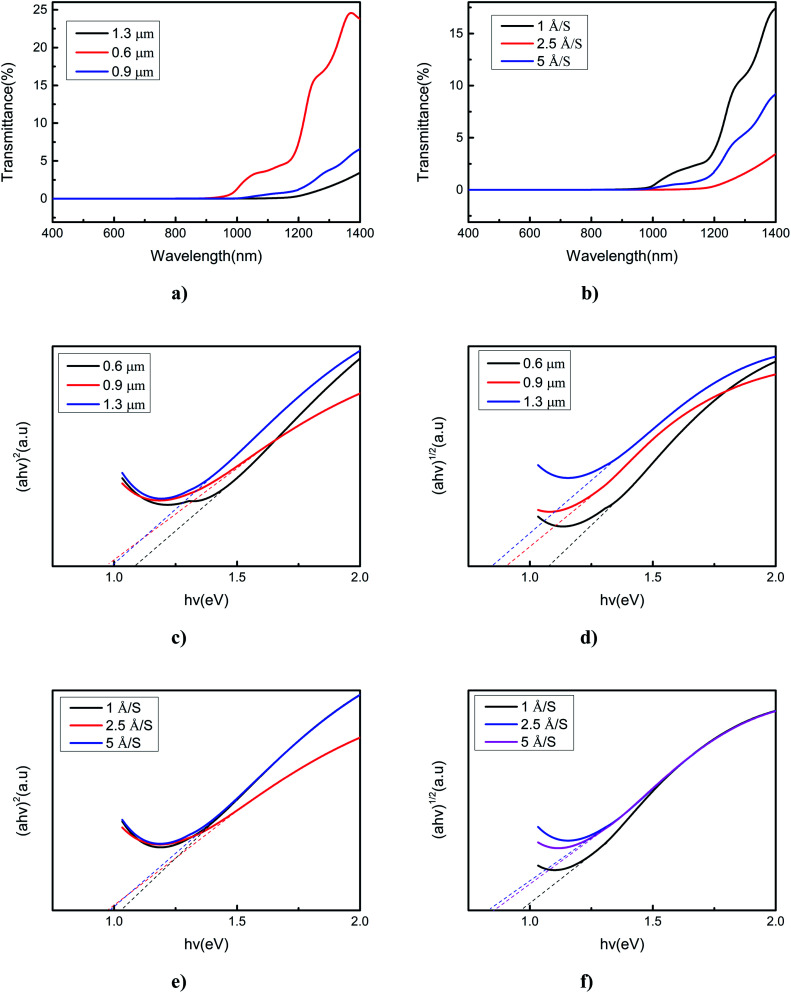
Optical curves of SnSe thin films with different deposition conditions: (a) transmittance of different film thicknesses with an evaporation rate of 2.5 Å S^−1^; (b) transmittance of different evaporation rates with a film thickness of 1.3 μm; (c) direct band gap of different film thicknesses with an evaporation rate of 2.5 Å S^−1^; (d) indirect band gap of different film thicknesses with an evaporation rate of 2.5 Å S^−1^; (e) direct band gap of different evaporation rates with a film thickness of 1.3 μm; (f) indirect band gap of different evaporation rates with a film thickness of 1.3 μm.

Optical transmittance has an important role in semiconductors because it reflects the absorption coefficient and band gap energy. Transmittance suddenly drops off near the absorption edge due to band-to-band transitions. Therefore, the band gap of a semiconductor can be calculated based on the value of transmittance, which can enable this material to be applied in different devices. The optical properties of SnSe thin films deposited under different conditions were studied using a Cary5000 UV-VIS-NIR spectrophotometer. For photons of wavelength between 400 and 1400 nm, transmittance of 0.01–24.8% (deposited with different film thicknesses) and 0.01–17.4% (deposited with different evaporation rates) were obtained. Transmittance varied with different film thicknesses and evaporation rates (Fig. 4a and b). This observation was because, as the film thickness and evaporation rate changed, the strain changed because the influence of the mismatch between glass and SnSe lattice implied generation of defects in the SnSe layer close to the glass substrate. From Fig. 4 we can conclude that, photons with a wavelength of 400–1400 nm, SnSe thin film of thickness of 1.3 μm, and evaporation rate of 2.5 Å S^−1^ obtained the lowest transmission of 0.01–4.8%. We used the transmittance data to calculate the absorption coefficient and optical band gap. The absorption coefficient (a) can be obtained from the transmittance and eqn (1)1
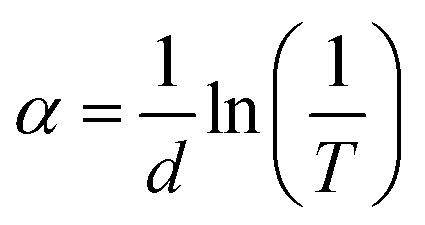
where *d* is the path length, *T* is the transmittance of the SnSe thin film, and *α* is the absorption coefficient. The band gap of SnSe can be obtained by eqn (2)

2 (*αhν*)^*n*^ = *A*(*hν* − *E*_g_)

where the value of *E*_g_ is dependent upon the type of band transition of the material. The band gap obtained when *n* is 1/2 represents the indirect band gap (Davis–Mott model). The band gap obtained when *n* is 2 represents the direct band gap (Tauc's model).^24^Fig. 4c and e show the relationship between Tauc's curve (*αhν*)^2^ and the photon energy (*hν*) for determining the direct band gap of different film thicknesses and evaporation rates, respectively. Fig. 4d and f show the relationship between the Davis–Mott curve (*αhν*)^1/2^ and the photon energy (*hν*) to determine the indirect band gap of different film thicknesses and evaporation rates, respectively. Fig. 4c and d reveal that the value of the direct band gap *E*_g_ of SnSe deposited with different film thicknesses was 0.98–1.13 eV and the value of the indirect band gap *E*_g_ was 0.87–1.13 eV. Besides, Fig. 4e and f show the value of the direct band gap to be 0.98–1.12 eV and indirect band gap to be 0.87–0.97 eV. The change of direct and indirect band gap was due to the difference in stacking faults and lattice defects as the film thickness or evaporation rate changes.^25^ The obtained values for the band gap are in accordance with theoretical expectations given by Singh and colleagues.^26^ Our results are in good agreement with those of reports of SnSe films prepared using other methods.^27^

**Fig. 5:**
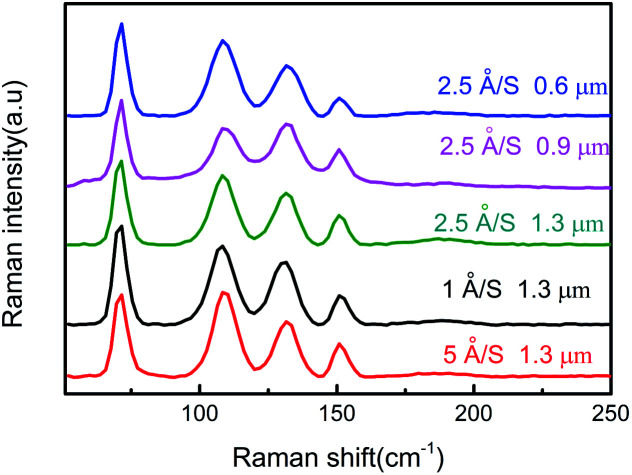
Raman spectra of the prepared SnSe thin films under different deposition conditions.

The optical properties of SnSe thin films were analyzed further by Raman spectroscopy. The excitation wavelength of the Raman spectrum was 542 nm, and the measurement range was 50 to 250 cm^−1^. Fig. 5 shows the Raman spectra of prepared SnSe thin films with different film thicknesses and evaporation rates. All Raman spectra show vibration modes at 74, 105.8, 130, and 152 cm^−1^, which correspond to A^1^_g_, B_3g_, A^2^_g_ and A^3^_g_ modes, respectively,^28,29^ and which conform closely to the characteristic pattern of SnSe. A_g_ and B_3g_ are two rigid shear modes of the layer relative to its adjacent layers, and they determine the characteristic planar vibration modes of the orthorhombic phase of SnSe. When considering the relationship between band gap energy and the Raman spectrum, all films obtained were suitable for photovoltaic applications.

### 3.4 Electrical properties

The electrical properties of SnSe thin films were measured by the Hall-effect measurement system using the van der Pauw method and four-point probes at room temperature. Table 3 shows the electrical properties of the SnSe thin films with different film thicknesses and evaporation rates. All films were p-type semiconductors and electrical properties varied with different film thicknesses and evaporation rates. Although the resistivity of the SnSe thin film deposited with a film thickness of 1.3 μm and evaporation rate of 2.5 Å S^−1^ was slightly higher than that under other deposition conditions, it exhibited the highest electron mobility (36.65 cm^2^ V^−1^ s^−1^). The highest electron mobility obtained in this condition may have been due to the better crystallinity and large crystallite size which may, ultimately, decrease inter-crystalline barriers. Also, high carrier mobility is required for improving the efficiency of solar-cell conversion. Hence, the SnSe film (obtained with a film thickness of 1.3 μm and evaporation rate of 2.5 Å S^−1^) with higher carrier mobility may be more appropriate for application in photovoltaic devices.

**Table 3:** Electrical properties of SnSe thin films with different film thicknesses and evaporation rates

Film thickness (μm)	Evaporation rate (Å S^−1^)	Resistivity (Ω cm)	Electron mobility (cm^2^ V^−1^ s^−1^)	Type of conduction	Carrier concentration (cm^−3^)
0.6	2.5	0.51	6.88	p	1.76 × 10^24^
0.9	2.5	1.96	15.63	p	2.04 × 10^23^
1.3	2.5	1.92	36.65	p	8.84 × 10^22^
1.3	1	0.89	13.63	p	5.09 × 10^23^
1.3	5	0.81	14.79	p	5.23 × 10^23^

### 3.5 *J*–*V* curves

**Fig. 6:**
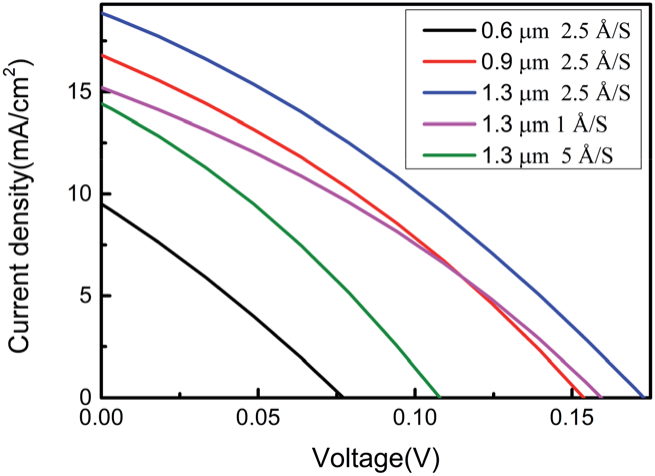
*J*–*V* characteristics of SnSe thin-film solar cells under different deposition conditions.

Fig. 6 shows the *J*–*V* curves measured for SnSe thin-film solar cells prepared under different conditions under AM 1.5 G sun-irradiation condition at room temperature. The film thickness and evaporation rate exhibit had a significant effect upon the power-conversion efficiency of the SnSe thin-film solar cell due to the effects on crystalline structure, microstructure, electrical properties and optical properties. Table 4 shows the device parameters of the fabricated solar cells. The devices fabricated with SnSe thin films were deposited and showed the highest efficiency (*η*) of 1.02% with a *V*_oc_ of 172 mV, short-circuit current density (*J*_sc_) of 18.87 mA cm^−2^, and fill factor (FF) of 31.27% with a film thickness of 1.3 μm and evaporation rate of 2.5 Å S^−1^. The FF was almost identical for a solar cell with a film thickness of 1.3 μm and evaporation rate of 2.5 Å S^−1^ compared with SnSe thin-film solar cells deposited under different conditions. However, *V*_oc_ and *J*_sc_ were improved obviously (Table 4). The improvement in *V*_oc_ and *J*_sc_ for the sample of film thickness 1.3 μm and evaporation rate of 2.5 Å S^−1^ was attributed mainly to the better crystallinity, larger grain size, and higher carrier mobility obtained in this work.

**Table 4:** Device parameters of fabricated solar cells

Film thickness (μm)	Evaporation rate (Å S^−1^)	*V*_oc_ (mV)	*J*_sc_ (mA cm^−2^)	FF (%)	*η* (%)
0.6	2.5	77	8.88	26.77	0.18
0.9	2.5	153	16.80	31.59	0.83
1.3	2.5	172	18.87	31.27	1.02
1.3	1	159	15.21	31.81	0.78
1.3	5	107	13.45	31.13	0.45

## 4. Conclusions

The structure, optical properties, morphology and electrical properties of SnSe thin films with different film thicknesses and evaporation rates were investigated. The crystallinity and grain size of SnSe thin films could be altered by changing the film thickness and evaporation rate. A larger grain size was obtained for SnSe thin films of film thickness 1.3 μm and evaporation rate of 2.5 Å S^−1^. A direct band gap of SnSe thin films of 0.98–1.13 eV and indirect band gap of SnSe thin films of 0.87–1.13 eV were obtained. All films exhibited p-type conductivity, and a relatively low resistivity of 1.92 Ω cm and maximum electron mobility of 36.65 cm^2^ V^−1^ s^−1^ were obtained with a film thickness of 1.3 μm and evaporation rate of 2.5 Å S^−1^. All films had an orthogonal structure and preferential (111) planar orientation. The manufactured SnSe solar cells exhibited a high power-conversion efficiency of 1.02%.

## Conflicts of interest

There are no conflicts to declare.

## Supplementary Material
